# Synopsis of the ticks of Algeria with new hosts and localities records

**DOI:** 10.1186/s13071-022-05424-2

**Published:** 2022-08-27

**Authors:** Noureddine Mechouk, Andrei Daniel Mihalca, Georgiana Deak, Zihad Bouslama

**Affiliations:** 1 Laboratory of Ecology of Terrestrial and Aquatics Systems (EcoSTAq), Department of Biology, Faculty of Science, Badji Mokhtar University, BP 12, 23200 Annaba, Algeria; 2 Department of Parasitology and Parasitic Diseases, University of Agricultural Sciences and Veterinary Medicine of Cluj-Napoca, Calea Mănăștur 3-5, 400372 Cluj-Napoca, Romania; 3 National Environmental Research Center, Sidi Amar Campus, Sidi Amar Campus, BP No. 2024, 23005 Annaba, Algeria

**Keywords:** Algeria, Ticks, Argasidae, Ixodidae, Geographical distribution, Host associations

## Abstract

**Background:**

Ticks are obligate hematophagous arthropods with a world-wide distribution that are extremely important not only in terms of human and animal health but also economically. In Algeria, information on tick species is scarce.

**Methods:**

A systematic literature review was performed using online databases. The information extracted from the databases was was supplemented by information from an original study. Ticks were collected from various hosts and by flagging from January 2018 to December 2019.

**Results:**

To date, in Algeria a total of 36 valid tick species belonging to two families have been recorded: (1) family Argasidae, with three *Argas* species and nine *Ornithodoros* species recorded; and (ii) family Ixodidae, with one *Dermacentor* species, three *Haemaphysalis* species, 10 *Hyalomma* species, four *Ixodes* species and six *Rhipicephalus* species recorded. The geographical distribution for each species was determined and listed. Eight new tick-host associations were recorded: four for *Ixodes inopinatus* sensu Estrada-Peña et al. 2014, one for *Rhipicephalus bursa*, one for *R. turanicus,* one for *Hyalomma marginatum* and one for *Hy. lusitanicum*. To our best knowledge, this study is the first to report the presence of *I. inopinatus* sensu Estrada-Peña et al. 2014 in Algeria. We also report here for the first time all tick species (Argasidae and Ixodidae) known to be present in Algeria.

**Conclusion:**

This article represents a tool for students and scientists who work in the field of ticks and provides important new data on the distribution of ticks in Algeria.

**Graphical Abstract:**

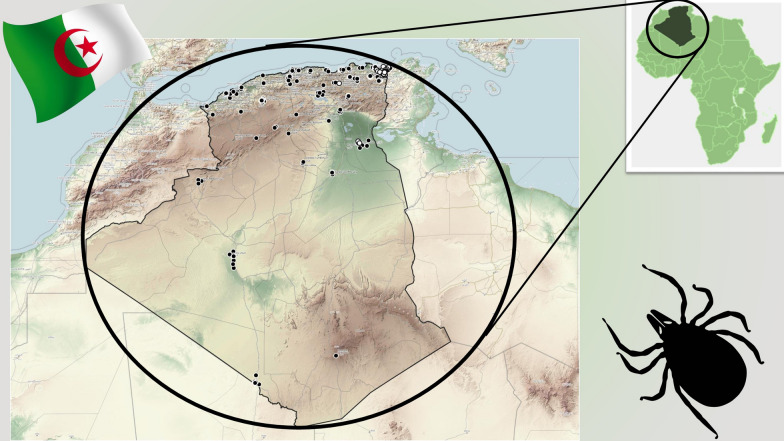

**Supplementary Information:**

The online version contains supplementary material available at 10.1186/s13071-022-05424-2.

## Background

Ticks are hematophagous arthropods that represent major potential hazards to human and animal health [1, 2]. There are over 900 tick species worldwide, divided into three families: the Ixodidae (hard ticks), Argasidae (soft ticks) and Nutalliellidae [3]. Ticks feed on various vertebrate hosts, passing through three active developmental stages (larva, nymph, adult). Depending on the behavior of each species, they may parasitize one, two or three hosts during a life-cycle. Ticks have a worldwide geographic distribution that is conditioned by biotic (temperature and humidity) and abiotic (host) factors. As a result, ticks are predisposed to harboring several types of microorganisms, including bacteria, viruses and parasites, and are therefore closely associated with the emergence of vector-borne diseases. Ticks play an important role in human and animal health as potential transmitters of a range of pathogens and can be the cause of significant economic losses. Therefore, ticks represent an important subject of research.

A total of 91 tick species have been reported in the Palearctic region, of which 67 species have been recorded in Europe and North Africa [4, 5]. Algeria is the largest country in Africa, the largest country in the Mediterranean Basin, and the tenth largest country in the world, with a surface area equivalent to 56% of the European Union’s (EU) total land area. Surprisingly, despite its vastness, habitat and climate diversity, little data are currently available on the diversity and distribution of tick fauna in this country. The first published research on ticks dates back to the first half of the twentieth century when Senevet [6] and Senevet and Rossi [7] studied the distribution of cattle ticks and the cattle tick–host association. More recently, several studies have been conducted on ticks and tick-borne diseases in Algeria [8–13], but significant knowledge gaps remain. We have therefore perfomed a systematic literature review and used the extracted data to update the knowledge on the diversity, geographical distribution and host association of tick species present in Algeria. We also highlight curent knowledge gaps to promote more targeted research.

## Methods

### Systematic literature review

The systematic literature review was performed according to the PRISMA 2020 protocol [14]. The search queries were done in online databases (PubMed, Google Scholar and Science Direct). Key terms searched in the title, abstract and/or keywords of studies were: “ticks AND Algeria,” “tiques AND Algérie,” “Ixodidae AND Algeria,” “Ixodidae AND Algérie,” “Argasidae AND Algeria” and “Argasidae AND Algérie”, which allowed the inclusion of both English and French literature. To ensure that all publications were included in the search, we also performed reverse reference tracking. Inclusion criteria were: (i) study area was within the territory of Algeria; and (ii) the data included the exact or approximate location of tick collection. No exclusion criteria were applied. From each paper, the following data were extracted (if available): tick species, stage, host species, sample size, prevalence, location with georeferenced information (decimal degree coordinate), the precision of the location (on a scale of 1–4, with 1 indicating that exact coordinates were known, 2 indicating that locality was known; 3 indicating that region/county was known; 4 indicating that location was unknown) and bibliographic source. The reported binomial names of tick species were updated to those currently accepted, according to the most recent taxonomical opinions [4].

All data were entered into a tabular database system (Microsoft Excel; Microsoft Corp., Redmond, WA, USA). All raw data are provided in Additional file 1: Table S1. The digital maps were made using QGIS version 3.14.

### Original data

In addition to using data extracted from the literature, we collected ticks from various animal hosts and by flagging in several localities of Algeria from January 2018 to December 2019. These data are also available in Additional file 1: Table S1. All collected ticks were preserved in 70% ethanol. The collected specimens were separated by developmental stage and sex and identified to species level using morphological characteristics according to Estrada-Peña et al. [15].

## Results

**Fig. 1:**
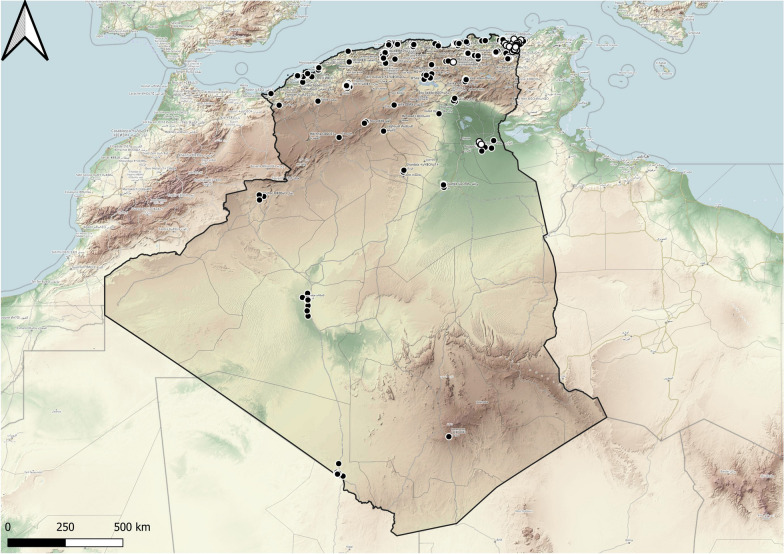
Distribution data on all members of family Ixodidae in Algeria: literature data (1922-present) are shown as black ovals and original data as white ovals

**Fig. 2:**
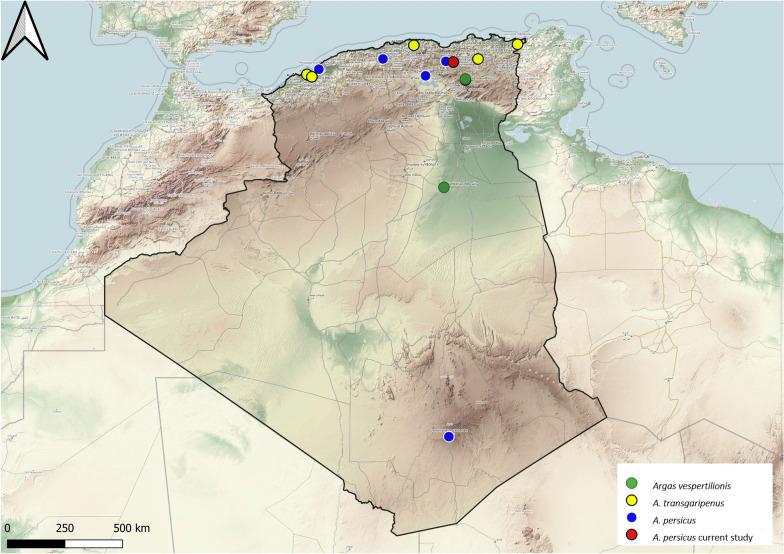
Geographical distribution of the Genus *Argas* in Algeria. Colored ovals show locations where there are records of *Argas persicus*, *A. transgariepinus* and *A. vespertilionis*, respectively

**Fig. 3:**
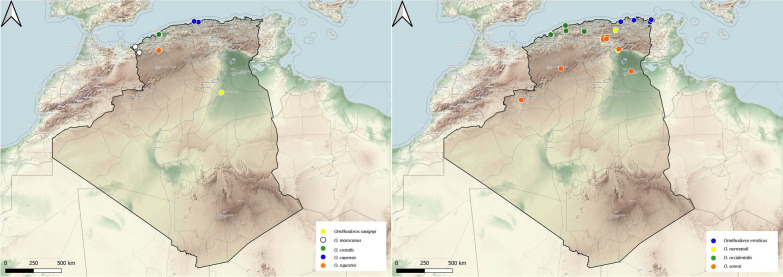
Geographical distribution of the genus *Ornithodoros* in Algeria. Colored ovals show locations where there are records of *Ornithodoros capensis*, *O. savignyi*, *O. costalis*, *O. rupestris*, *O. marocanus*, *O. erraticus*, *O. normandi*, *O. occidentalis* and *O. sonrai*

**Fig. 4:**
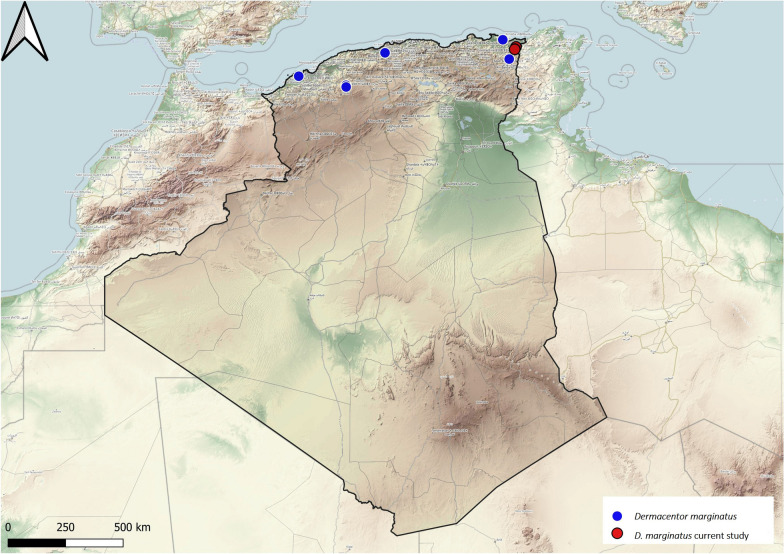
Geographical distribution of *Dermacentor marginatus* in Algeria

**Fig. 5:**
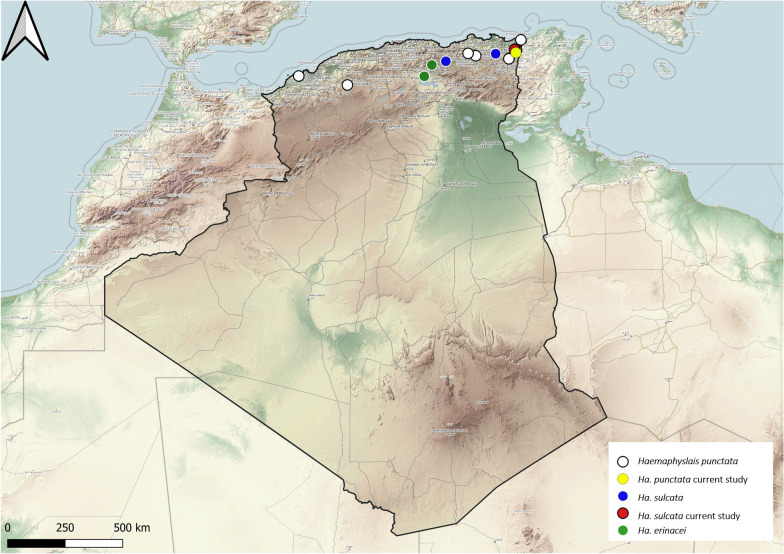
Geographical distribution of the genus *Haemaphysalis* in Algeria. Colored ovals show locations where there are records of *Haemaphysalis erinacei*, *Ha. punctata* and *Ha. sulcata*, respectively

**Fig. 6:**
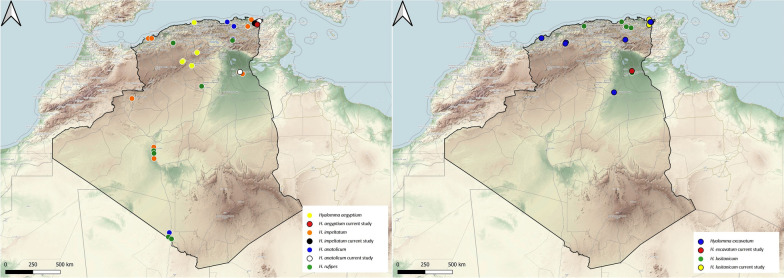
Geographical distribution of the genus *Hyalomma* in Algeria. Colored ovals show locations where there are records of *Hyalomma aegyptium*, *Hy. anatolicum*, *Hy. dromaderii*, *Hy. excavatum*, *Hy. impletatum* and *Hy. lusitanicum*

**Fig. 7:**
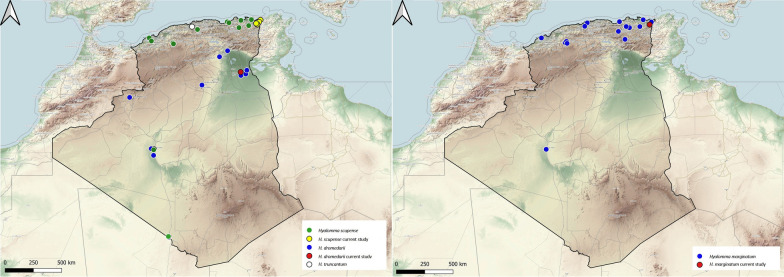
Geographical distribution of the genus *Hyalomma* in Algeria. Colored ovals show locations where there are records of *Hyaloma marginatum,* *Hy. rufipes*, *Hy. scupense* and *Hy. truncantum*

**Fig. 8:**
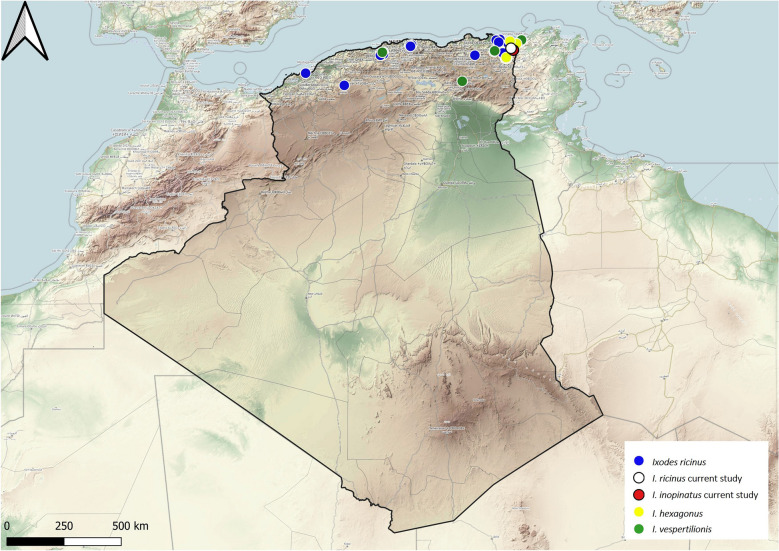
Geographical distribution of the genus *Ixodes* in Algeria. Colored ovals show locations where there are records of *Ixodes hexagonus*, *I. inopinatus* sensu Estrada-Peña et al. 2014, *I. ricinus* and *I. vespertilionis*

**Fig. 9:**
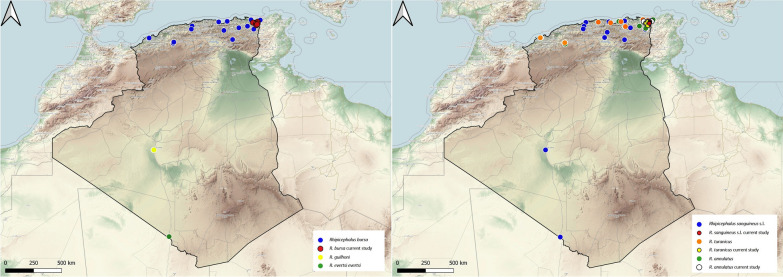
Geographical distribution of the Genus *Rhipicephalus* in Algeria. Colored ovals show locations where there are records of *Rhipicephalus annulatus*, *R. bursa*, *R. evertsi evertsi*, *R. guilhoni*, *R. sanguineus* sensu lato, *R. turanicus*

Overall, the database resulting from this study includes 171,929 individual ticks, in 36 species (12 Argasidae and 24 Ixodidae) in a total of 853 records. The distribution maps for each tick species are shown in Figs. 1–9, respectively, and discussed in detail in the Discussion. The overview of tick-host associations and the synoptic list of ticks are shown in Tables 1 and 2.

**Table 1:** Synoptic list of ticks and their hosts reported in Algeria (1922-present)

Tick species	Host and/or locality	Stage	References
*Argas persicus*	*Gallus gallus domesticus*	A	[12, 55]
		n/a	[56]
	Environment	A	[55]
		n/a	[57]
*Argas transgariepinus*	*Eptesicus isabellinus*	A, L	[36]
	*Hypsugo savii*	L	[36]
*Argas vespertilionis*	*Plecotus gaisleri*	L	[36]
	*Tadarida aegyptiaca*	L	[36]
*Ornithodoros capensis*	*Larus michahellis* (nests)	A	[58]
		n/a	[59] [10] [56]
	Sea birds (nests)	n/a	[57]
*Ornithodoros costalis*	Burrows (natural)	n/a	[18]
*Ornithodoros erraticus*	Rodent burrows	n/a	[10] [57] [56]
*Ornithodoros marocanus*	Burrows (natural)	n/a	[18]
*Ornithodoros normandi*	Burrows (natural)	n/a	[18]
*Ornithodoros occidentalis*	Rodent burrows	n/a	[10] [57] [56]
*Ornithodoros rupestris*	Rodent burrows	n/a	[10] [57] [56]
*Ornithodoros savignyi*	*Camelus dromedarius*	n/a	[60]
*Ornithodoros sonrai*	Rodent burrows	n/a	[10] [57] [56]
*Dermacentor marginatus*	*Bos taurus*	A	[44] [61] 62]
		n/a	[63]
	*Sus scrofa*	A	[11] and current study
		n/a	[49]
*Haemaphysalis erinacei*	*Paraechinus aethiopicus*	n/a	[45]
	*Atelerix algirus*	n/a	[28]
*Haemaphysalis punctata*	*Bos taurus*	A	[44] [61] [12] and current study
		n/a	[64] [65]
	*Canis familiaris*	n/a	[66]
	*Ovis aries*	A	[12] and current study
	*Sus scrofa*	A	[11]
	Livestock	n/a	[67]
*Haemaphysalis sulcata*	*Bos taurus*	A	[37] and current study
	*Capra aegagrus hircus*	A	[12]
	*Ovis aries*	A	[12] and current study
*Hyalomma aegyptium*	*Testudo graeca*	A	[68] [12] [69] and current study
		A, N, L	[70]
		n/a	[71] [72]
	Livestock	n/a	[67]
	*Bos taurus*	n/a	[6]
*Hyalomma anatolicum*	*Bos taurus*	n/a	[64] [63]
		A	[11] [39] and current study
	*Camelus dromedarius*	n/a	[43]
		A	Current study
	*Capra aegagrus hircus*	n/a	[38]
*Hyalomma dromedarii*	*Camelus dromedarius*	n/a	[43] [73] [74] [13]
		A	Current study
	*Ovis aries*	n/a	[25]
	*Pipistrellus kuhlii*	A	[36]
*Hyalomma excavatum*	*Bos taurus*	A	[44] [61] [62] [37]
		n/a	[63, 64]
		N	[37]
	*Camelus dromedarius*	n/a	[13]
		A	Current study
	*Equus cabalus*	A	[41]
*Hyalomma impeltatum*	*Bos taurus*	A	[44] [62] and current study
		n/a	[38]
	*Camelus dromedarius*	n/a	[43] [73] [74] [13]
	*Equus cabalus*	A	[41]
	*Ovis aries*	A	[25]
		n/a	[38]
*Hyalomma lusitanicum*	*Bos taurus*	n/a	[6] [75] [64] [65]
		A	[44] [61] [62] [63] [39] and current study
	*Camelus dromedarius*	A	Current study
	*Capra aegagrus hircus*	A	[11]
	*Equus cabalus*	A	[41]
	*Ovis aries*	A	[11]
*Hyalomma marginatum*	*Atelerix algirus*	A	Current study
	*Bos taurus*	A	[44] [61] [8] [62] [37] [39] and current study
		n/a	[64] [65] [63]
	*Capra aegagrus hircus*	A	[11]
		n/a	[38]
	*Carduelis carduelis*	A, N	[59]
	*Equus cabalus*	A	[41]
	Flagging	A, N, L	[48]
	*Ovis aries*	A	[8]
		n/a	[25] [38]
	*Sus scrofa*	n/a	[49]
		A	Current study
*Hyalomma rufipes*	*Bos taurus*	n/a	[6] [63]
		A	[61]
	*Camelus dromedarius*	n/a	[43] [73]
*Hyalomma scupense*	*Bos taurus*	n/a	[6] [76] [75] [64] [65] [37]
		A	[44] [61] [8] [62] [12] [39] and current study
	*Camelus dromedarius*	n/a	[43]
	*Capra aegagrus hircus*	n/a	[38]
	*Ovis aries*	n/a	[25] [38]
		A	[12]
	Livestock	n/a	[67]
	n/a	n/a	[77]
*Hyalomma truncatum*	*Camelus dromedarius*	n/a	[43] [74]
	*Bos taurus*	n/a	[63]
*Ixodes hexagonus*	*Atelerix algirus*	A	[11]
		n/a	[42]
	*Canis familiaris*	A	[11]
*Ixodes inopinatus* sensu Estrada-Peña et al. 2014	*Bos taurus*	A	Current study
	Flagging	A	Current study
	*Podarcis hispanica vaucheri*	L	Current study
	*Psammodromus algirus*	L	Current study
	*Timon pater*	N	Current study
*Ixodes ricinus*	*Atelerix algirus*	n/a	[46]
		A, N, L	Current study
	*Bos taurus*	A	[44] [61] [9] [12] and current study
		n/a	[74] [64] [65] [78] [63]
	*Canis familiaris*	n/a	[66]
		A	[12]
	*Capra aegagrus hircus*	n/a	[38]
	*Eptesicus isabellinus*	n/a	[79]
	Flagging	n/a	[74]
		N	[80] [48]
		L	[48]
		A	[80] [48] and current study
	*Hepestes ichneumon*	A	[11]
	*Ovis aries*	A	[11]
		n/a	[38]
	*Pipistrellus kuhlii*	n/a	[79]
	*Plegadis falcinellus*	n/a	[81]
	*Podarcis hispanica vaucheri*	n/a	[82]
		N	[83]
		L	[83] and current study
	*Psammodromus algirus*	n/a	[82]
		N, L	[83] and current study
	*Rattus rattus*	A	[84]
	*Rhinolophus hipposideros*	A, L	[36]
	*Sus scrofa*	n/a	[49]
	*Tadarida teniotis*	A, L	[36]
	*Timon pater*	n/a	[82]
		N	[83]
		L	[83] and current study
	Livestock	n/a	[67]
*Ixodes vespertilionis*	*Miniopterus schreibersii*	L	[85]
		n/a	[79]
	*Myotis cappaccinii*	A	[36]
		L	[36] [85]
	*Myotis emarginatus*	A, L	[36]
	*Myotis punicus*	A	[36]
		L	[36] [85]
	*Rhinolophus blasii*	L	[85]
		n/a	[79]
	*Rhinolophus euryale*	L	[85]
	*Rhinolophus ferrumequinum*	A	[85]
		n/a	[79]
*Rhipicephalus annulatus*	*Bos taurus*	n/a	[6] [75] [64] [38]
		A	[44] [61] [62] [12] [39] [37] amd current study
		N, L	[37]
	*Canis familiaris*	A	[12] and current study
	*Capra aegagrus hircus*	A	[12] and current study
		n/a	[38]
	*Equus cabalus*	A	[12] and current study
		n/a	[38]
	*Ovis aries*	A	[12] and current study
		n/a	[38]
	Livestock	n/a	[67]
*Rhipicephalus bursa*	*Atelerix algirus*	A	[12] and current study
		n/a	[42]
	*Bos taurus*	n/a	[6] [75] [64] [65] [78] [63]
		A	[44] [61] [8] [11] [12] [62] [37] [39] and current study
		N	[37]
	*Canis familiaris*	A	[12] [50] and current study
	*Capra aegagrus hircus*	A	[11]
		n/a	[38]
	*Equus cabalus*	A	[41]
	*Felis catus*	A	[12] and current study
		N	Current study
	*Ovis aries*	A	[11] [12] and current study
		n/a	[38]
	*Sus scrofa*	A	Current study
	Livestock	n/a	[67]
*Rhipicephalus evertsi*	*Camelus dromedarius*	n/a	[43]
	*Ovis aries*	n/a	[25]
*Rhipicephalus guilhoni*	*Ovis aries*	n/a	[25]
*Rhipicephalus sanguineus* sensu lato	*Atelerix algirus*	A	[8] [11] [12] and current study
		n/a	[45] [46]
	*Bos taurus*	n/a	[6] [65] [63]
		A	[44] [11] [62] [12] [39] and current study
	*Camelus dromedarius*	A	[43]
	*Canis aureus*	A	[11]
	*Canis familiaris*	A	[11] [66] [12]; [50] and current study
	*Capra aegagrus hircus*	A	[8] [11] [12]
		n/a	[38]
	*Felis catus*	A	[12] and current study
	Flagging	A	[80] [48]
		N, L	[48]
	*Hypsugo savii*	n/a	[86]
	*Hepestes ichneumon*	A	[11]
	*Miniopterus schreibersii*	n/a	[86]
	*Myotis cappaccinii*	n/a	[86]
	*Myotis punicus*	n/a	[86]
	*Ovis aries*	n/a	[25] [38]
		A	[11] [12] and current study
	*Paraechinus aethiopicus*	n/a	[45]
	*Sus scrofa*	A	[11]
	Livestock	n/a	[67]
*Rhipicephalus turanicus*	*Atelerix algirus*	A	[8] and current study
		n/a	[42]
		N	Current study
	*Bos taurus*	A	[44] [61] [8] [37] and current study
		n/a	[75] [64]
		N	[37]
	*Canis familiaris*	A	[50] and current study
	*Capra aegagrus hircus*	A	[8]
	*Felis catus*	A	Current study
	*Sus scrofa*	A	[49]
	Flagging	A, N, L	[48]

*A* Adults, *N* nymphs, *L* larvae; *n/a* not specified/unknown

**Table 2:** Synoptic list of hosts and their ticks reported in Algeria (1922-present)

Host/Locality	Tick species
**Mammalia**	
* Atelerix algirus*	*Haemaphysalis erinacei* (n/a), *Hyalomma marginatum* (A), *Ixodes hexagonus* (A), *Ixodes ricinus* (A, N, L), *Rhipicephalus bursa* (A), *Rhipicephalus sanguineus* sensu lato (A), *Rhipicephalus turanicus* (A)
* Paraechinus aethiopicus*	*Haemaphysalis erinacei* (n/a), *Rhipicephalus sanguineus* sensu lato (n/a)
* Bos taurus*	*Dermacentor marginatus* (A), *Haemaphysalis punctata* (A), *Haemaphysalys sulcata* (A), *Hyalomma anatolicum* (A), *Hyalomma detritum (scupense)* (A), *Hyalomma excavatum* (A, N), *Hyalomma impeltatum*, *Hyalomma lusitanicum*, *Hyalomma marginatum, Hyalomma rufipes* (A), *Ixodes inopinatus* sensu Estrada-Peña et al. 2014 (A), *Ixodes ricinus* (A), *Rhipicephalus annulatus* (A, N, L), *Rhipicephalus bursa* (A, N), *Rhipicephalus sanguineus* sensu lato (A), *Rhipicephalus turanicus* (A, N), *Hyalomma truncatum* (n/a)
* Camelus dromedarius*	*Hyalomma anatolicum* (A), *Hyalomma scupense* (n/a), *Hyalomma dromedarii* (A), *Hyalomma excavatum* (A), *Hyalomma impeltatum* (A), *Hyalomma rufipes* (n/a), *Hyalomma lusitanicum* (A), *Hyalomma truncatum* (n/a), *Rhipicephalus evertsi evertsi* (n/a), *Rhipicephalus sanguineus* sensu lato (n/a), *Ornithodoros savignyi* (n/a)
* Canis aureus*	*Rhipicephalus sanguineus* sensu lato (A)
* Canis familiaris*	*Haemaphysalis punctata* (n/a), *Ixodes hexagonus* (A), *Ixodes ricinus* (A), *Rhipicephalus annulatus* (A), *Rhipicephalus bursa* (A), *Rhipicephalus sanguineus* sensu lato (A), *Rhipicephalus turanicus* (A)
* Capra aegagrus hircus*	*Haemaphysalys sulcata* (A), *Hyalomma anatolicum* (A), *Hyalomma lusitanicum* (A), *Hyalomma marginatum* (A), *Hyalomma scupense* (n/a), *Ixodes ricinus* (n/a), *Rhipicephalus annulatus* (A), *Rhipicephalus bursa* (A), *Rhipicephalus* sanguineus sensu lato (A), *Rhipicephalus turanicus* (A)
* Equus cabalus*	*Hyalomma excavatum* (A), *Hyalomma impeltatum* (A), *Hyalomma lusitanicum* (A), *Hyalomma marginatum* (A), *Rhipicephalus annulatus*(A), *Rhipicephalus bursa*(A)
* Felis catus*	*Rhipicephalus bursa* (A, N), *Rhipicephalus sanguineus* sensu lato (A), *Rhipicephalus turanicus* (A)
* Mangoose (Hepestes ichneumon)*	*Rhipicephalus sanguineus* sensu lato (A), *Ixodes ricinus* (A)
* Ovis aries*	*Haemaphysalis punctata* (A), *Haemaphysalys sulcata* (A), *Hyalomma scupense* (A), *Hyalomma dromedarii* (n/a), *Hyalomma impeltatum* (n/a), *Hyalomma lusitanicum* (A), *Hyalomma marginatum* (A), *Ixodes ricinus* (A), *Rhipicephalus annulatus* (A), *Rhipicephalus bursa* (A), *Rhipicephalus evertsi evertsi* (n/a), *Rhipicephalus guilhoni* (n/a), *Rhipicephalus sanguineus* sensu lato (A)
* Sus scrofa*	*Dermacentor marginatus* (A), *Haemaphysalis punctata* (A), *Hyalomma marginatum* (A), *Ixodes ricinus* (A), *Rhipicephalus bursa* (A) *Rhipicephalus sanguineus* sensu lato (A), *Rhipicephalus turanicus* (n/a)
* Eptesicus Isabellinus*	*Argas transgariepinus* (A, L), *Ixodes ricinus* (n/a)
* Hypsugo savii*	*Argas transgariepinus* (L), *Rhipicephalus sanguineus* sensu lato (n/a)
* Miniopterus schreibersii*	*Ixodes vespertilionis* (L), *Rhipicephalus sanguineus* sensu lato (n/a)
* Myotis cappaccinii*	*Ixodes vespertilionis* (A, L), *Rhipicephalus sanguineus* sensu lato (n/a)
* Myotis emarginatus*	*Ixodes vespertilionis* (A, L)
* Myotis punicus*	*Ixodes vespertilionis* (A, L), *Rhipicephalus sanguineus* sensu lato (n/a)
* Pipistrellus kuhlii*	*Hyalomma dromedarii* (A), *Ixodes ricinus* (n/a)
* Plecotus gaisleri*	*Argas vespertilionis* (L)
* Rhinolophus blasii*	*Ixodes vespertilionis* (L)
* Rhinolophus euryale*	*Ixodes vespertilionis* (L)
* Rhinolophus ferrumequinum*	*Ixodes vespertilionis* (A)
* Rhinolophus hipposideros*	*Ixodes ricinus* (A, L)
* Tadarida teniotis*	*Ixodes ricinus* (A, L)
* Tadarida aegyptiaca*	*Argas vespertilionis* (L)
* Rattus rattus*	*Ixodes ricinus* (A)
**Reptilia**	
* Podarcis hispanica vaucheri*	*Ixodes inopinatus* sensu Estrada-Peña et al. 2014 (L), *Ixodes ricinus* (N, L)
* Psammodromus algirus*	*Ixodes inopinatus* sensu Estrada-Peña et al. 2014 (L), *Ixodes ricinus* (N, L)
* Timon pater*	*Ixodes inopinatus* sensu Estrada-Peña et al. 2014 (N), *Ixodes ricinus* (N, L)
* Testudo graeca*	*Hyalomma aegyptium* (A, N, L)
Aves	
* Carduelis carduelis*	*Hyalomma marginatum* (A, N)
* Gallus gallus domesticus*	*Argas persicus* (A)
* Plegadis falcinellus*	*Ixodes ricinus* (n/a)
**Locality**	
Animal Shelters	*Argas persicus* (n/a)
Poultry House	*Argas persicus* (A)
Rodent Burrows	*Ornithodoros erraticus* (n/a), *Ornithodoros occidentalis* (n/a), *Ornithodoros rupestris* (n/a), *Ornithodoros sonrai* (n/a)

Underlined tick species represent new host-tick associations for Algeria

*A* Adult, *N* Nymph, *L* Larvae, *n/a* not known

###  Literature data

Overall, data were extracted from 56 papers (Additional file 1: Table S1), yielding 728 unique records, with a total of 168,429 ticks in 35 species, of which 12 species were members of family Argasidae (*Argas persicus*, *A. transgariepinus*, *A. vespertilionis*, *Ornithodoros capensis*, *O. costalis*, *O. erraticus*, *O. marocanus*, *O. normandi*, *O. occidentalis*, *O. rupestris*, *O. savignyi*, *O. sonrai*) and 23 species were members of family Ixodidae (*Dermacentor marginatus*, *Haemaphysalis erinacei*, *Ha. punctata*, *Ha. sulcata*, *Hyalomma aegyptium*, *Hy. anatolicum*, *Hy. dromedarii*, *Hy. excavatum*, *Hy. impeltatum*, *Hy. lusitanicum*, *Hy. marginatum*, *Hy. rufipes*, *Hy. scupense*, *Hy. truncatum*, *Ixodes hexagonus*, *I. ricinus*, *I. vespertilionis*, *Rhipicephalus annulatus*, *R. bursa*, *R. evertsi evertsi*, *R. guilhoni*, *R. sanguineus* sensu lato, *R. turanicus*).

### Original data

A total of 3500 ticks were recovered from 13 hosts belonging to 18 tick species (Table 1). Of these, *Ixodes inopinatus* sensu Estrada-Peña et al. 2014 is reported for the first time in Algeria. Eight new tick-host associations are also reported.

## Discussion

The tick fauna of Algeria has been reported in several historical studies as well as in more recent papers. However, there have been no studies on the ticks of southern Algeria, and only 26 records (3.05%) of all reported records are from latitudes below 30°N. Most records (*n* = 617; 72.33%) are from the northern part of the country (between 35°N and 36°N) (Fig. 1). This suggests a huge gap in knowledge on tick diversity and distribution across most of Algeria’s territory. Our study reports eight new tick-host associations for Algeria. We also report for the first time the presence of *I. inopinatus* sensu Estrada-Peña et al. 2014 in Algeria.

### Genus *Argas*

Three *Argas* species have been reported in Algeria. *Argas persicus* was reported only on a few occasions and only from domestic poultry. Most records are from the north of the country, with only one record from the south (Fig. 2). This is consistent with the ecology of the species, which is known to be an endophilic tick present in the desert, temperate Mediterranean regions and rainforests [16].

The two other *Argas* species recorded in Algeria are both bat specialists. *Argas transgariepinus* was reported from two vespertilionid bats in the northern part of the country (Fig. 2). *Argas vespertilionis* is an endophilic tick that was reported in Algeria in bats of belonging to the genera* Plecotus* and* Tadarida*, respectively (Fig. 2)

### Genus *Ornithodoros*

Nine species belonging to this genus have been found in Algeria, most of them parasitic on wild birds. *Ornithodoros capensis* is a nesting tick species that specializes on seabirds [17], with a worldwide distribution in the Pacific, Atlantic and Indian Oceans and in East Africa's Rift Valley. In Algeria, *O. capensis* has been found infesting nests of the seabird *Larus michahellis* (Fig. 3).

The range of *O. savignyi* in Africa is relatively wide, extending across most of the continent’s regions [16]. In Algeria, this tick was found on camels (Fig. 3).

The *O. erraticus* complex is a group of species comprising nine species of ticks: *O. occidentalis*,* O. costalis*,* O. rupestris*,* O. kairouanensis*,* O. meriones*,* O. erraticus*,* O. marocanus*,* O. sonrai* and *O. normandi* [18]. In Africa, species of this complex have been collected in Algeria, Morocco, Tunisia, Mauritania, Senegal, Gambia, Mali, Burkina Faso, Niger, Benin, Togo, Ivory Coast, Guinea, Guinea Bissau, Liberia, Chad and Cameroon [18]. In Algeria, seven species of this group have been found infesting nests of the seabird *Larus michahellis*, natural burrows and rodents. Regarding their range, *O. costalis, O. rupestris*,* O. marocanus* have been collected in northwest Algeria, *O. erraticus, O. normandi* and *O. occidentalis* have been collected in northeast Algeria and *O. sonrai* has a wider distribution (Fig. 3).

### Genus *Dermacentor*

*Dermacentor marginatus* is the only species of this genus recorded in Algeria. Adult ticks feed on sheep, cattle, goats and dogs, and larvae and nymphs parasitize small mammals, mainly rabbits, and birds [16]. In North Africa, *D. marginatus* shares the same habitat as *Ixodes ricinus* [16]. In Algeria, *D. marginatus* ticks were reported on two mammal species distributed in the country's northern region, mainly at high altitudes (Fig. 4), suggesting a co-distribution relationship with wild boar.

### Genus *Haemaphysalis*

Three species of genus *Haemaphysalis* were reported from Algeria, with most reports mentioning *Ha. sulcata* and *Ha. punctata*; there is only a single report of *Ha. erinacei*, collected from the desert hedgehog *Paraechinus aethiopicus* and the North African hedgehog *Atelerix algirus*, occurring in the northern part of the country, in a steppe area (Fig. 5).

*Haemaphysalis punctata* is a three-host tick which parasitizes cattle and sheep and occasionally also horses, goats and antelopes. This tick is distributed throughout Europe, North Africa and East Asia [16]. In Algeria, it was found on cattle, but infestations on sheep, wild boars and dogs have been reported. It is present in the northern part of the country (Fig. 5).

*Haemaphysalis sulcata* is also a three-host tick, with sheep being the most common host. This tick occurs over a wide range in North Africa, Europe and Asia [19–21]. In Algeria, it is found on sheep, cattle and goats and is localized in the country's northeastern part (Fig. 5).

### Genus *Hyalomma*

This genus is the most diversified in terms of the number of species reported from Algeria and 10 species of genus *Hyalomma* have been reported in Algeria. *Hyalomma aegyptium* is a three-host tick with tortoises of genus *Testudo* being the main hosts for all developmental stages. This tick is present in the Mediterranean basin and in the Black Sea [22]. In Algeria, *Testudo greaca* is the main and only reported host for *Hy. aegyptium*. This tick has been reported in the northern and interior regions of the country, as its distribution is dependent on the presence of its host, as shown in previous studies [23] (Fig. 6).

*Hyalomma anatolicum* is an endophilic tick with a two- or three-host life-cycle. Large ungulates, mainly cattle, horses, camels, sheep and goats are hosts for all developmental stages [24]. This tick is widely distributed throughout Africa and Asia [21]. In Algeria, *Hy. anatolicum* is reported on livestock and is present in the northern, interior and southern regions of the country (Fig. 6).

*Hyalomma dromedarii* has a two- or three-host life-cycle. The camel is the preferred host, but domestic mammals can also harbor this tick. *Hyaloma dromedarii* occurs in Mediterranean, steppe and desert climates [16]. It is reported mainly on camels in Algeria, but infestations on sheep have been reported in Algeria [25]. The presence of *Hy. dromedarii* is strictly associated with the geographical distribution of its main host, the dromedary (Fig. 6).

*Hyalomma excavatum* is a two- or three-host exophilic tick, with domestic mammals being frequent hosts but cattle and camels being the main ones. Insectivores, lagomorphs and rodents are the preferred hosts of the immature stages. This tick species is known to occur in North Africa, East Africa, southern Europe, the Middle East and Central Asia [21, 24]. In Algeria, it is reported from livestock (Fig. 6).

*Hyalomma impeltatum* has a two- or three-host life-cycle. Members of family Camelidae and family Bovidae are the common hosts for adults, while those of Leporidae (rabbits and hares) and Muridae (small rodents) are common hosts for immature stages [4]. *Hyalomma impeltatum* is widespread in the Palearctic region [26]. In Algeria, it is reported on livestock, with the dromedary as the most common host (Fig. 6).

*Hyalomma lusitanicum* is a three-host tick feeding on cattle and other domestic and wild ungulates. It is restricted to the western part of the Mediterranean sub-region of the Palearctic zoogeographical region [27]. In Algeria, it has been collected from five mammalian hosts (Fig. 6).

*Hyalomma marginatum* is a two-host tick with mammals as the primary hosts. Its geographical distribution includes southern Europe and North Africa [28]. In Algeria, *Hy. marginatum* is the most reported species of its genus (Fig. 7).

*Hyalomma rufipes* is a two-host exophilic tick. Adults feed on cattle, sheep, goats, horses and camels. Larvae and nymphs infest birds and leporids [27]. In Algeria, it seems to have a broad range (Fig. 7).

Adult and immature stages of *Hy. scupense* feed primarily on cattle and horses but there may have been rare sightings on sheep, donkeys, pigs, camels and wild ungulates [29]. *Hyalomma scupense* has a broad distribution, ranging from North Africa and Western Europe to the eastern parts of China [30]. In Algeria, cattle are reported as the preferred host of *Hy. scupense*, but infestations on other domestic ungulates have also been reported (Fig. 7).

Domestic herbivores are the preferred hosts of the adult of *Hy. truncatum*, while immature stages parasitize hares and rodents [16]. This tick species has been reported in the northern and southern parts of Algeria infesting cattle and camels (Fig. 7).

### Genus *Ixodes*

Four species of the genus *Ixodes* were reported in Algeria. *Ixodes hexagonus* is a three-host species with carnivorous mammals and hedgehogs as the main hosts. It has a wide distribution in Europe [31], but in Algeria only two reports are available on this tick, collected from dogs and hedgehogs (Fig. 8).

*Ixodes inopinatus* sensu Estrada-Peña et al. 2014 is an exophilic tick. The immature stages feed on lizards and adults feed on red foxes and sheep [32, 33]. Earlier studies reported its presence in Spain, Portugal, Tunisia and Morocco [32], but more recently it has also been reported in Eastern Europe and Tunisia, in sympatry with *I. ricinus* [33, 34]. In Algeria, prior to our report, no data were available regarding *I. inopinatus* sensu Estrada-Peña et al. 2014 [32], as previously any specimens may have been misidentified as *I. ricinus*. In the current study, immature stages were collected from lizards while adults were collected from cattle, providing new host association records for the country. It is important to note that we collected this species in a forest habitat (*Quercus* spp.). Its distribution range in Algeria is the northeastern region (Fig. 8). However, given the lack of surveys, we assume it is more widespread than we expect and may be spread at least over the northern region of the country.

*Ixodes ricinus* is a three-host tick with catholic behavior. Immature stages parasitize birds and lizards, while mammals are the preferred hosts for adults [15]. This tick is present in the Western Palearctic region in terms of its range, with over 300 host species. In Algeria, the reported hosts include nine mammals and three reptile species. The adult stage has been found on mammals and the immature mainly on lizards. Its distribution is limited to the northern part of Algeria, mainly in the mountainous regions (Fig. 8).

*Ixodes vespertilionis* is a three-host, endophilic tick that parasitizes bats and is widely distributed in Europe [35]. In northern Algeria, previous reports have reported infestation by tick of troglodyte bats [36] (Fig. 8).

### Genus *Rhipicephalus*

The genus *Rhipicephalus* is represented in Algeria by six species, all of which are fairly abundant in most of the domestic hosts. This tick feeds on the same host during all developmental stages, with the preferred hosts being ruminants, but infestations of wild boar and cats are also frequently reported [16]. In Algeria, *R. annulatus* is mainly located in the country's northern region which is characterized by a dominant Mediterranean climate. Previous studies have shown that cattle are the main host in Algeria, but infestations on other mammals (dogs, horses, goats and sheep) have also been reported [12, 37–39] (Fig. 9).

*Rhipicephalus bursa* is a two-host tick that feeds on various mammals. The geographical distribution of *R. bursa* extends around the Mediterranean Sea [40]. In Algeria, it has been collected from cattle, sheep, goats, horses, dogs, cats and hedgehogs [11, 12, 41, 42]. This report reveals its presence on wild boars for the first time, constituting a new host association for Algeria. Its distribution is limited to the northern region of the country (Fig. 9).

*Rhipicephalus evertsi evertsi* is a two-host tick that feeds on cattle and wild ungulates and has a teletropic feeding behavior. This tick is widespread throughout African countries, with a preference for the Afrotropical zoogeographic region [40]. In Algeria, *R. evertsi evertsi* was reported sporadically on sheep and camels in the southern part of the country by Bouhous et al. [25, 43]; however, these authors suggested that it could be an accidental infestation (Fig. 9).

*Rhipicephalus guilhoni* has a three-host life-cycle, with adult ticks infesting mammals (horses, cattle, sheep, dogs and wild carnivores) and immature stages feeding on small mammals. This tick has a range in Africa that extends from Senegal to Ethiopia [40]. Only one report is known for Algeria, with ticks collected on sheep, located in the south of the country [25] (Fig. 9).

*Rhipicephalus sanguineus* sensu lato (brown dog tick) is the most widespread tick in terms of its host spectrum in Algeria, which includes 15 mammalian species [8, 11, 12, 44–46]. It is a three-host tick that feeds mainly on dogs but can be found on other hosts [47]. In Algeria, *R. sanguineus* s.l. has been found in domestic fauna (dogs, camels, goats, cattle, cats and sheep) and wildlife (jackal, bats, hedgehog, wild boar and mongoose). In this study, we report it in cattle, dogs, sheep, cats and hedgehogs. Only the adult stage was reported from the hosts, while the immatures were collected by flagging [48]. The brown dog tick has a worldwide distribution. In Algeria, it is present in the northern, central and southwestern regions of the country (Fig. 9).

*Rhipicephalus turanicus* is present in the Palearctic region, although its actual distributional boundaries remain unclear due to its ambiguous phylogeny [4]. This species is a three-host tick. In Algeria, it is present in the northern part of the country where it infests cattle, goats, dogs, wild boars and hedgehogs [8, 37, 42, 49, 50] Interestingly, we collected it from cats, which is a new host association for Algeria (Fig. 9).

To summarize, due to the global changes that have taken place in recent years, Algeria is facing desertification [51]. The absence of ecological barriers between Algeria and neighboring countries, the legal and illegal movement of animals in the North African region and the different types of livestock farming practiced in Algeria (e.g. nomadism, pastoralism, and transhumance) are factors potentially responsible for a high diversity and geographical distribution of ticks. However, from the data presented in this article, it is clear that there are several gaps in data regarding tick diversity and distribution in Algeria. Despite the high wild terrestrial vertebrate diversity in this country (111 mammals, 406 birds and 99 reptiles) [52–54], there are surprisingly few studies on their ticks. Moreover, vast territories of the country remain completely unstudied for ticks. Future approaches to fill these gaps can reveal the presence of so far unreported tick species for Algeria.

## Conclusions

This study is the first to report the presence of *Ixodes inopinatus* sensu Estrada-Peña et al. 2014 in Algeria and provides valuable and important new important data on the distribution of ticks and new tick-host associations.

## Supplementary Information

**Additional file 1: Table S1.** Data analyzed in the current study.

## Data Availability

The datasets supporting the conclusions are included within the paper and its additional file. The ticks are stored in the collection of the USASMV Cluj-Napoca and are available from the corresponding author on reasonable request.

## References

[1] Parola P, Raoult D (2001). Ticks and tick borne bacterial diseases in humans: an emerging infectious threat. Clin Infect Dis.

[2] Jongejan F, Uilenberg G (2004). The global importance of ticks. Parasitology.

[3] Barker SC, Murrell A. Systematics and evolution of ticks with a list of valid genus and species names. In: Bowman AS, Nuttall P, editors. Ticks: biology, disease and control. Cambridge: Cambridge University Press; 2008. p. 1–39.

[4] Guglielmone AA, Robbins RG, Apanaskevich DA, Petney TN, Estrada-Peña A, Horak IG (2014). The hard ticks of the world (Acari: Ixodida: Ixodidae).

[5] Mihalca AD, Estrada-Peña A, Petney TN. Introduction. In: Mihalca AD, Estrada-Peña A, Petney TN, editors. Ticks of Europe and North Africa. Cham: Springer; 2018. p. 1–3.

[6] Senevet G. Contribution à l’étude des Ixodidés (IX° note)—Espèces trouvées en Algérie sur les bovins pendant les mois d’été. Arch Inst Pasteur Afr Nord. 1922;2:519–28.

[7] Senevet G, Rossi G. Contribution à l’étude des Ixodidés (XII° note). Étude saisonnière des Ixodidés de la région de Bouira (Algérie): Arch Inst Past Alg. 1924;2:223–32.

[8] Bitam I, Parola P, Matsumoto K, Rolain JM, Baziz B, Boubidi SC (2006). First molecular detection of *R. conorii*, *R. aeschlimannii*, and *R. massiliae* in ticks from Algeria. Ann N Y Acad Sci.

[9] Dib L, Bitam I, Bensouilah M, Parola P, Raoult D (2009). First description of *Rickettsia monacensis* in *Ixodes ricinus* in Algeria. Clin Microbiol Infect.

[10] Lafri I, Leulmi H, Baziz-Neffah F, Lalout R, Mohamed C, Mohamed K (2015). Detection of a novel *Rickettsia sp* in soft ticks (*Acari:* Argasidae) in Algeria. Microbes Infect.

[11] Leulmi H, Aouadi A, Bitam I, Bessas A, Benakhla A, Raoult D (2016). Detection of *Bartonella tamiae*, *Coxiella burnetii* and *rickettsiae* in arthropods and tissues from wild and domestic animals in Northeastern Algeria. Parasit Vectors.

[12] Boucheikhchoukh M, Laroche M, Aouadi A, Dib L, Benakhla A, Raoult D (2018). MALDI-TOF MS identification of ticks of domestic and wild animals in Algeria and molecular detection of associated microorganisms. Comp Immunol Microbiol Infect Dis.

[13] Bellabidi M, Benaissa MH, Bissati-Bouafia S, Harrat Z, Brahmi K, Kernif T (2020). *Coxiella burnetii* in camels (*Camelus dromedarius*) from Algeria: Seroprevalence, molecular characterization, and ticks (Acari: Ixodidae) vectors. Acta Trop.

[14] Page MJ, McKenzie JE, Bossuyt PM, Boutron I, Hoffmann TC, Mulrow CD, The PRISMA (2020). statement: an updated guideline for reporting systematic reviews. BMJ.

[15] Estrada-Peña A, Mihalca AD, Petney TN (2018). Ticks of Europe and North Africa: a guide to species identification.

[16] Walker AR. Ticks of domestic animals in Africa: a guide to identification of species. Edingburgh: Biosciences Reports; 2003.

[17] Hoogstraal H, Clifford CM, Keirans JE (1979). The Ornithodoros capensis group (Alectorobius) of the Palaearctic and (Acarina: Ixodoidea: Argasidae) oriental regions. *O (A) coniceps* identity, bird and mammal hosts, virus infections, and distribution. J Parasitol.

[18] Trape JF, Diatta G, Arnathau C, Bitam I, Sarih M, Belghyti D (2013). The epidemiology and geographic distribution of relapsing fever borreliosis in West and North Africa, with a review of the *Ornithodoros erraticus* complex (Acari: Ixodida). PLoS ONE.

[19] Pomerantzev BI. Ixodid ticks (Ixodidae), fauna of the USSR. New series 41: Arachnoidea. Paukoobraznye 4(2).

[20] Bouattour A, Darghouth MA, Daoud A (1999). Distribution and ecology of ticks (Acari: Ixodidae) infesting livestock in Tunisia: an overview of eighth years field collections. Parassitologia.

[21] Estrada-Peña A, Bouattour A, Camicas JL, Walker AR (2004). Ticks of domestic animals in the mediterranean region. A guide to identification of species.

[22] Široký P, Petrželková KJ, Kamler M, Mihalca AD, Modrý D (2006). *Hyalomma aegyptium* as dominant tick in tortoises of the genus *Testudo* in Balkan countries, with notes on its host preferences. Exp App Acarol.

[23] Mihalca AD, Dumitrache MO, Magdaş C, Gherman CM, Domşa C, Mircean V (2012). Synopsis of the hard ticks (Acari: Ixodidae) of Romania with update on host associations and geographical distribution. Exp App Acarol.

[24] Bakheit MA, Latif A, Vatansever Z, Seitzer U, Ahmed J, Mehlhorn H (2012). The huge risks due to *Hyalomma* ticks. Arthropods as vectors of emerging diseases.

[25] Bouhous A, Aissi M, Harhoura K (2011). Prevalence of Ixodidae in sheep brought for slaughter in Adrar municipal abattoir Southwest Algeria. Sci Parasitol.

[26] Apanaskevich DA, Horak IG. The genus *Hyalomma Koch*, 1844. IX. Redescription of all parasitic stages of *H*. (*Euhyalomma*)* impeltatum* Schulze & Schlottke, 1930 and* H*. (*E.*)* franchinii* Tonelli Rondelli, Acari: Ixodidae. Syst Parasitol. 2009;73:199–218. 10.1007/s11230-009-9190-x 19472079

[27] Apanaskevich DA, Santos-Silva MM, Horak IG, The genus* Hyalomma* Koch, 1844. IV. Redescription of all parasitic stages of *H*. (*Euhyalomma*) *lusitanicum* Koch, 1844 and the adults of *H*. (*E*.)* franchinii* Tonelli Rondelli, 1932 (Acari: Ixodidae) with a first description of its immature stages. Folia Parasitol. 1844;2008:61–74. 10.14411/fp.2008.009 18578168

[28] Apanaskevich DA, Horak IG (2008). The genus *Hyalomma Koch*, 1844: v re-evaluation of the taxonomic rank of taxa comprising the *H.* (*Euhyalomma*)* marginatum* Koch complex of species (Acari: Ixodidae) with redescription of all parasitic stages and notes on biology. Int J Acarol.

[29] Bursali A, Keskin A, Tekin S (2012). A review of the ticks (Acari: Ixodida) of Turkey: species diversity, hosts and geographical distribution. Exp Appl Acarol.

[30] Gharbi M, Darghouth MA (2014). A review of *Hyalomma scupense* (Acari, Ixodidae) in the Maghreb region: from biology to control. Parasite.

[31] Kolonin GV. Fauna of ixodid ticks of the world (Acari, Ixodidae). 2009. http://www.kolonin.org/. Accessed 22 Feb 2015/

[32] Estrada-Peña A, Nava S, Petney T (2014). Description of all the stages of *Ixodes inopinatus* n. sp. (Acari: Ixodidae). Ticks Tick-Borne Dis.

[33] Chitimia-Dobler LR, Rieß O, Kahl S, Wölfel G, Dobler S, Nava E-PA (2018). *Ixodes inopinatus* occurring also outside the Mediterranean region. Ticks Tick Borne Dis.

[34] Younsi H, Fares W, Cherni S, Dachraoui K, Barhoumi W, Najjar C, Zhioua E (2020). *Ixodes inopinatus* and *Ixodes ricinus* (Acari: Ixodidae) are sympatric ticks in North Africa. J Med Entomol.

[35] Hornok S, Kontschán J, Kováts D, Kovács R, Angyal D, Görföl T, et al. Bat ticks revisited: *Ixodes ariadnae* sp. nov and allopatric genotypes of I *vespertilionis* in caves of Hungary. Parasit Vectors. 2014;7:202. 10.1186/1756-3305-7-202. 10.1186/1756-3305-7-202 PMC4029976 24766822

[36] Bendjeddou ML, Loumassine HA, Scheffler I, Bouslama Z, Amr Z (2017). Bat ectoparasites (Nycteribiidae, Streblidae, Siphonaptera, Heteroptera, Mesostigmata, Argasidae, and Ixodidae) from Algeria. J Vector Ecol.

[37] Bouchama B, Dik B, Benia F, Mouffok C (2020). Dynamique Saisonnière Des Tiques (Acari: Ixodidae) Parasites Des Bovins Dans La Région Semi-Aride De La Wilaya De Sétif Algérie. Bull Soc Zool Fr.

[38] Sadeddine R, Diarra AZ, Laroche M, Mediannikov O, Righi S, Benakhla A (2020). Molecular identification of protozoal and bacterial organisms in domestic animals and their infesting ticks from north-eastern Algeria. Ticks Tick-Borne Dis.

[39] Lotfi D, Karima K (2020). Identification incidence of hard tick species during summer season 2019 in Jijel Province (northeastern Algeria). J Parasit Dis.

[40] Walker JB, Keirans JE, Horak IG (2000). The genus Rhipicephalus (Acari: Ixodidae). Guide to the brown ticks of the world.

[41] Kouidri M, Mohammed S, Al-Abidine KZ (2019). First study on the composition species of tick (Ixodidae) infesting horses in Algeria. Agricultura.

[42] Senaoui C, Boukheroufa M, Sakraoui F, Sakraoui W (2020). Preferential fixation sites and relative frequencies of ectoparasites at *Atelerix algirus* (Lereboullet, 1842) in a locality on the North East of Algeria. Ecol Environ Conserv.

[43] Bouhous A, Aissi M, Harhoura KH (2008). Etude des Ixodidae chez le dromadaire dans le sud algérien, région d’Adrar. Ann Med Vet.

[44] Yousfi-Monod R, Aeschlimann A (1986). Recherches sur les tiques (Acarina, Ixodidae), parasites de bovidés dans l’Ouest Algérien: 1. Inventaire systématique et dynamique saisonnière. Ann Parasitol Hum Comp.

[45] Khaldi M, Socolovschi C, Benyettou M, Barech G, Biche M, Kernif T (2012). Rickettsiae in arthropods collected from the North African Hedgehog (*Atelerix algirus*) and the desert hedgehog (*Paraechinus aethiopicus*) in Algeria. Comp Immunol Microbiol Infect Dis.

[46] Becir F, Chetoui MB, Bitam I, Bouslama Z (2015). *Atelerix Algirus* Ectoparasites of El-Kala National Park (Algeria). Int Proc Chem Biol Environ Eng (IPCBEE).

[47] Dantas-Torres F (2012). Biology and ecology of the brown dog tick *Rhipicephalus sanguineus*. Parasit Vectors.

[48] Belabed AI, Zediri H, Shehab A, Bouslama Z (2015). The effect of altitude on seasonal dynamics of Ticks (Acari: Ixodida) in Northeastern Algeria. Adv Environ Biol.

[49] Zeroual F, Bitam I, Ouchene N, Leulmi H, Aouadi A, Benakhla A (2014). Identification and seasonal dynamics of ticks on wild boar (*Sus scrofa*) in the extreme north-east of Algeria. Bulla Soc Zool Fr.

[50] Kebbi R, Nait-Mouloud M, Hassissen L, Ayad A (2019). Seasonal activity of ticks infesting domestic dogs in Bejaia province Northern Algeria. Onderstepoort J Vet Res.

[51] Nedjraoui D, Bédrani S (2008). La désertification dans les steppes algériennes : causes, impacts et actions de lutte. Vertigo.

[52] Ahmim M. Les Mammiferes Sauvages D'algerie Répartition et Biologie de la Conservation. HAL Id: hal-02375326; 2019.

[53] Isenmann P, Moali A. Birds of Algeria. Paris SEOF; 2000. ISBN: 9782950654885.

[54] Beddek Menad. Déficit de connaissances de la biodiversité et biologie de la conservation: le cas de l’herpétofaune d’Algérie. PhD thesis. Montpellier: Université Montpellier; 2017.

[55] Ouchene N, Nebbak A, Ouchene-Khelifi NA, Dahmani A, Zeroual F, Khelef D (2020). Molecular detection of avian spirochete *Borrelia anserina* in *Argas persicus* ticks in Algeria. Comp Immunol Microbiol Infect Dis.

[56] Lafri I, Benredjem W, Neffah-Baaziz F, Lalout R, Abdelouahed K, Gassen B (2018). Inventory and update on argasid ticks and associated pathogens in Algeria. New Microbes New infect.

[57] Lafri I, El Hamzaoui B, Bitam I, Leulmi H, Lalout R, Mediannikov O (2017). Detection of relapsing fever *Borrelia* spp, *Bartonella* spp. and anaplasmataceae bacteria in argasid ticks in Algeria. PlOs Negl Trop Dis.

[58] Baaziz Neffah F, Kernif T, Beneldjouzi A, Boutellis A, Morsli A, Harrat Z (2014). *Carios capensis* (ACARI: ARGASIDAE) in the nests of the yellow legged Gull (Larus Michahellis) in the Agueli island of Reghaia. Algeria Int J Bot Res.

[59] Baziz-Neffah F, Bitam I, Kernif T, Beneldjouzi A, Boutellis A, Berenger JM (2015). Contribution à la connaissance des ectoparasites d’oiseaux en Algérie. Bull Soc Zool Fr.

[60] Chalon G (1923). Présence d’*Ornithodoros savignyi* (Audouin) à Ouargla (Sahara algérien). Bull Soc Pathol Exot.

[61] Boulkaboul A (2003). Parasitisme des tiques (Ixodidae) des bovins à Tiaret, Algérie. Rev Elev Med Vet Pays Trop.

[62] Mokhtaria K, Ammar AA, Ammar SSM, Chahrazed K, Fadela S, Belkacem BT (2018). Survey on species composition of Ixodidae tick infesting cattle in Tiaret (Algeria). Trop Agric.

[63] Rahal M, Medkour H, Diarra AZ, Bitam I, Parola P, Mediannikov O (2020). Molecular identification and evaluation of *Coxiella-like* endosymbionts genetic diversity carried by cattle ticks in Algeria. Ticks Tick-Borne Dis.

[64] Elfegoun MB, Gharbi M, Djebir S, Kohil K (2013). Dynamique d’activité saisonnière des tiques ixodidés parasites des bovins dans deux étages bioclimatiques du nord-est algérien. Rev Elev Med Vet Pays Trop.

[65] Elfegoun MB, Kohil K, Gharbi M, Afoutni L, Benachour ML (2019). Cinétique d’infestation par les tiques des bovins de la région subhumide de Constantine en Algérie. Rev Elev Med Vet Pays Trop.

[66] Matallah F, Benakhla A, Bouattour A (2013). Infestation du chien par *Rhipicephalus sanguineus* dans deux régions de l'extrême nord-est de l'Algérie. Rev Elev Med Vet Pays Trop.

[67] Sergent E, Poncet A (1937). Tableau de la répartition saisonnière des tiques les plus rèpandues en Algèrie. Arch Inst Pasteur Alger.

[68] Kautman M, Tiar G, Papa A, Široký P (2016). AP92-like Crimean-Congo hemorrhagic fever virus in *Hyalomma aegyptium* ticks, Algeria. Emerg Infect Dis.

[69] Benyahia H, Diarra AZ, Gherissi DE, Bérenger JM, Benakhla A, Parola P (2020). Molecular and MALDI-TOF MS characterisation of *Hyalomma aegyptium* ticks collected from turtles and their associated microorganisms in Algeria. Ticks Tick-Borne Dis.

[70] Tiar G, Tiar-Saadi M, Benyacoub S, Rouag R, Široký P (2016). The dependence of *H. yalomma aegyptium* on its tortoise host *Testudo graeca* in Algeria. Med Vet Entomol.

[71] Bitam I, Kernif T, Harrat Z, Parola P, Raoult D (2009). First detection of *Rickettsia aeschlimannii* in *Hyalomma aegyptium* from Algeria. Clin Microbiol Infect.

[72] Lakehal K, Saidi R, Mimoune N, Benaceur F, Baazizi R, Chaibi R, et al. The study of ectoparasites and mesoparasites in turtles (*Testudo graeca graeca*) in the region of Laghouat (south of Algeria). Bull Univ Agric Sci Vet Med Cluj Napoca. 2020;77:1.

[73] Djerbouh A, Kernif T, Beneldjouzi A, Socolovschi C, Kechemir N, Parola P (2012). The first molecular detection of *Rickettsia aeschlimannii* in the ticks of camels from southern Algeria. Ticks Tick-Borne Dis.

[74] Kernif T, Djerbouh A, Mediannikov O, Ayach B, Rolain JM, Raoult D (2012). *Rickettsia africae* in *Hyalomma dromedarii* ticks from sub-Saharan Algeria. Ticks Tick-Borne Dis.

[75] Benchikh-Elfegoun MC, Benakhla A, Bentounsi B, Bouattour A, Piarroux R (2007). Identification et cinétique saisonnière des tiques parasites des bovins dans la région de Taher (Jijel) Algérie. Ann Med Vet.

[76] Senevet G (1924). Description de la nymphe de *Hyalomma mauritanicum* Senevet 1922. Arch Inst Pasteur Alger.

[77] Sergent E, Donatien A, Parrot L, Lestoquard F. Cycle évolutif de *Theileria dispar* du bœuf chez la tique *Hyalomma mauritanicum*. Arch Inst Pasteur Alger. 1936;14:259–94.

[78] Dib L, Lafri I, Boucheikhchoukh M, Dendani Z, Bitam I, Benakhla A (2019). Seasonal distribution of *Rickettsia* spp. in ticks in northeast Algeria. New Microbes New Infect.

[79] Bendjoudi D, Yedou W, Beneldjouzi A, Mechouk N, Bendjeddou ML (2019). On Bat Ectoparasites (Nycteribiidae, Streblidae, Siphonaptera, Mesostigmata And Ixodidae) From Chrea National Park (Central Atlas Mountains) Algeria. Bull Soc Zool Fr.

[80] Benredjem W, Leulmi H, Bitam I, Raoult D, Parola P (2014). *Borrelia garinii* and *Rickettsia monacensis* in *Ixodes ricinus* ticks, Algeria. Emerg Infect Dis.

[81] Touati L, Figuerola J, Alfarhan AH, Samraoui B (2015). Distribution patterns of ectoparasites of Glossy Ibis (*Plegadis falcinellus*) chicks. Zool Ecol.

[82] Bouslama Z, Soualah-Alila H, Belabed A, Ouali K (2009). Etude du système Tiques-Lézard dans le parc national d’El Kala (Nord-Est algérie). Mésogée.

[83] Soualah-Alila H, Bouslama Z, Amr Z, Hani RB (2015). Tick infestations (Acari: Ixodidae) on three lizard species from Seraidi (Annaba District), northeastern Algeria. Exp Appl Acarol.

[84] Becir F, Bitam I, Hannachi H, Bouslama Z. *Rattus rattus* parasites of El-kala national park (Algeria), Chap. 38. Intech Open; 2012.

[85] Khelfaoui F, Kebaci A, Benyacoub S (2018). New data on Insecta and Acarina parasitizing bats (Mammalia: Chiroptera) in Numidia, eastern Algeria. Bull Soc Zool Fr.

[86] Bendjeddou ML, Bitam I, Abiadh A, Bouslama Z, Amr Z (2013). New records of arthropod ectoparasites of bats from North—Eastern Algeria. Jordan J Biol Sci.

